# Rapid CRISPR/Cas9 Editing of Genotype IX African Swine Fever Virus Circulating in Eastern and Central Africa

**DOI:** 10.3389/fgene.2021.733674

**Published:** 2021-08-30

**Authors:** Hussein M. Abkallo, Nicholas Svitek, Bernard Oduor, Elias Awino, Sonal P. Henson, Samuel O. Oyola, Stephen Mwalimu, Nacrya Assad-Garcia, Walter Fuchs, Sanjay Vashee, Lucilla Steinaa

**Affiliations:** ^1^Animal and Human Health Program, International Livestock Research Institute (ILRI), Nairobi, Kenya; ^2^Department of Synthetic Biology and Bioenergy, J. Craig Venter Institute, Rockville, MD, United States; ^3^Institute of Molecular Virology and Cell Biology, Friedrich-Loeffler-Institut, Federal Research Institute for Animal Health, Greifswald-Insel Riems, Germany

**Keywords:** ASFV, CRISPR/Cas9, gene editing, in-frame deletion, virus isolation

## Abstract

African swine fever virus (ASFV) is the etiological agent of a contagious and fatal disease of domestic pigs that has significant economic consequences for the global swine industry. Due to the lack of effective treatment and vaccines against African swine fever, there is an urgent need to leverage cutting-edge technologies and cost-effective approaches for generating and purifying recombinant virus to fast-track the development of live-attenuated ASFV vaccines. Here, we describe the use of the CRISPR/Cas9 gene editing and a cost-effective cloning system to produce recombinant ASFVs. Combining these approaches, we developed a recombinant virus lacking the non-essential gene A238L (5EL) in the highly virulent genotype IX ASFV (ASFV-Kenya-IX-1033) genome in less than 2 months as opposed to the standard homologous recombination with conventional purification techniques which takes up to 6 months on average. Our approach could therefore be a method of choice for less resourced laboratories in developing nations.

## Introduction

African swine fever virus (ASFV) is a large, enveloped, double-stranded DNA virus, which is the etiological agent of a contagious and lethal disease of domestic pigs that has significant economic ramifications for the global swine industry. The disease is endemic in most sub-Saharan African countries and presently ravaging the swine industry in Asia and Europe. Due to the lack of effective treatment and vaccines, African swine fever (ASF) control relies on containment measures such as stamping out by slaughter and disposal of all infected and potentially infected animals and strict biosecurity measures (Penrith and Vosloo, 2009).

The conventional technologies for producing ASFV mutants are based on homologous recombination involving replacement of viral genes with a reporter gene β-galactosidase (β-Gal), β-glucuronidase (GUS), or fluorescent markers (Rodríguez et al., 1992; Gómez-Puertas et al., 1995; Zsak et al., 1996; Neilan et al., 1997a; Afonso et al., 1998; Hernaez et al., 2006; Portugal et al., 2012; O’Donnell et al., 2015; Borca et al., 2017; Chen et al., 2020). An improvement on this system involves the combination of the Cre/loxP recombination system and a transfer plasmid with the β-glucoronidase gene (GUS), which then allows for the exchange of the target gene with the GUS reporter gene, facilitating multiple deletions in the genome, albeit sequentially (Abrams and Dixon, 2012). However, these approaches are limited by the rare and low-efficiency homologous recombination events as well as the laborious isolation of the recombinant virus through limiting dilutions. Consequently, there is an urgent need to deploy enabling technologies, such as Clustered Regularly Interspaced Short Palindromic Repeats (CRISPR)/CRISPR-associated nuclease 9 (Cas9) (Barrangou et al., 2007) to expedite development of ASFV vaccines.

The CRISPR/Cas9 system is a prokaryotic defense mechanism that confers resistance against invading phages and plasmids (Barrangou et al., 2007). Generally, upon introduction of double-strand breaks (DSB) in the genome by Cas9, two DNA repair pathways, the non-homologous end joining (NHEJ) and the homology-directed repair (HDR), come into play to fix the genome (Kanaar et al., 1998; Featherstone and Jackson, 1999). Despite its high efficiency in creating indel mutations, the NHEJ pathway has repeatedly been reported to result in in-frame deletions, which might result in semi-functional proteins (Morton et al., 2006; Qi et al., 2013), thus reducing knock-out (KO) efficiency. In contrast to NHEJ, the less efficient but high-fidelity HDR pathway relies on a repair (donor) template to retain precise genome integrity (Kanaar et al., 1998).

The CRISPR/Cas9 technology is now being utilized for versatile applications such as precise interrogation of basic biological functions (Hilton and Gersbach, 2015), development of biotechnology products (e.g., vaccines) (Okoli et al., 2018; Chang et al., 2019) and treatment of diseases (Morishige et al., 2019; Ajami et al., 2020). This technology can therefore be harnessed for ASFV functional genetics to elucidate the role of specific genes in virus replication processes, virus-host interactions, or virus virulence, and more importantly for accelerating vaccine development for ASF.

Three recent reports have shown the successful application of CRISPR/Cas9 in targeting the ASFV genome (Borca et al., 2018; Hübner et al., 2018; Woźniakowski et al., 2020). Hübner et al. (2018) used CRISPR/Cas9 as a strategy to confer resistance against ASFV infection by using a cell line expressing CRISPR/Cas9 and p30-specific guide RNA, and successfully showed that ASFV replication can be suppressed *in vitro*. Borca et al. (2018) presented the use of the CRISPR/Cas9 gene-editing/HDR system as a superior alternative to traditional homologous recombination in generating recombinant ASFV expressing an exogenous fluorescent marker, and Woźniakowski et al. (2020) attempted CRISPR/Cas9 modification of a field isolate of ASFV.

Despite the successful application of CRISPR/Cas9-mediated gene editing in ASFV (Borca et al., 2018), an affordable, time-saving, and efficient approach for isolation of recombinant viruses remains a challenge due to the many rounds of limiting dilution and plaque picking that is required to isolate the recombinant virus away from the wildtype virus before it can be tested for the desired phenotype. To speed up production of recombinant ASFV, Rathakrishnan et al. (2020) took advantage of fluorescent reporter markers and single-cell fluorescence-activated cell sorting (FACS). However, this system remains a practical challenge as the cell sorter needs to be installed in a biosafety cabinet at the level required for ASFV in the respective country, and it is costly thus may be a challenge in resource-constrained settings.

In this report, we describe the combination of CRISPR/Cas9-mediated HDR and a rapid and straightforward cloning strategy to generate ASFV deletion mutants in genotype IX ASFV (ASFV-Kenya-IX-1033), which circulates in eastern and central Africa. We chose to edit A238L (5EL), a non-essential ASFV gene known to control host immune evasion by inhibiting the activation of TNF-alpha by modulating NF-kB, NF-AT, and c-Jun trans-activation (Neilan et al., 1997b; Dixon et al., 2004; Granja et al., 2006; Silk et al., 2007). Our cloning approach combines limiting dilution, fluorescence microscopy, and a simple microscope object marker to reduce the overall time for generating deletion mutants to < 2 months. This cheaper alternative could therefore be a method of choice for laboratories in developing nations that do not have as many resources. Additionally, with this cloning technique, as is the case with the approach by Rathakrishnan et al. (2020), it is possible to use multiple fluorescent reporter genes concurrently, hence facilitating generation of multiple gene deletion recombinants. Our approach, combined with the efficient CRISPR/Cas9-mediated homologous recombination for gene deletions, can lead to the rapid production of recombinant ASFV for vaccine candidates and functional genetics studies. In addition, we explored the practicality of generating an ASFV deletion mutant through NHEJ.

## Materials and Methods

### Cells and Viruses

Wild boar lung cell line (WSL), kindly provided by the Collection of Cell Lines in Veterinary Medicine at the Friedrich-Loeffler-Institut, Greifswald, Insel Riems, Germany, was maintained in Dulbecco’s Modified Eagle Medium (DMEM, Sigma-Aldrich) supplemented with 10% FBS + penicillin/streptomycin (Roth) + Glutamine (Roth).

ASFV-Kenya-IX-1033 and dsRed-expressing ASFV-Kenya-IX-1033, herein referred to as ASFV-Ke and ASFV-Ke-dsRed, respectively, have been reported previously (Hübner et al., 2018). Reporter gene expression by the respective virus recombinants was detected by fluorescence microscopy (Optika).

This study has been reviewed and approved by the international livestock research institute (ILRI) institutional biosafety committee (IBC).

### Dose-Response Curve for Antibiotic Selection of WSL Cells

To determine the minimum concentration of puromycin dihydrochloride (Sigma-Aldrich) required to kill non-transfected WSL cells, 2 × 10^5^ cells/well were seeded into 6-well plates and incubated overnight at 37°C with 5% CO_2_. On the following day, the complete growth medium was replaced with selection medium supplemented with a range of puromycin dihydrochloride concentrations (0, 0.5, 1, 2, 4, 6, 8, and 10 μg/ml). The cells were monitored daily using a light microscope to observe the percentage of surviving cells. The medium was replaced every 3 days with freshly prepared selection medium containing the range of puromycin dihydrochloride concentrations being tested.

### Establishment of Cas9-Expressing Cells and Western Blot Analyses

To generate an ASFV-permissive cell line expressing Cas9 nuclease, 2 μg of pSpCas9(BB)-2A-Puro (PX459) (Ran et al., 2013) plasmid was transfected into a wild boar lung cell line WSL (Portugal et al., 2012; Keil et al., 2014; Hübner et al., 2018) using the Lipofectamine 3000 reagent (Invitrogen) as per the manufacturer’s instructions. Forty-eight hours after the transfection, selection media (DMEM + 10% FBS + penicillin/streptomycin + glutamine) containing 1 μg/ml of puromycin dihydrochloride was used to select for cells co-expressing Cas9 and puromycin resistance. Puromycin-resistant WSL cells were cloned in 96-well plates and confirmed for Cas9 expression using Western blot. Briefly, proteins from WSL and WSL-Cas9 lysates were separated on SDS-PAGE and blotted on nitrocellulose membrane. The mouse α-CRISPR/Cas9 mAb (clone: 7A9-3A3, Cat. No. SAB4200701; Sigma), and the β-tubulin specific monoclonal (T4026, Sigma) were used at dilutions of 1:250 and 1: 1,000, respectively. The blots were subsequently incubated for 1 h at room temperature with goat anti-mouse IgG(L + H)-HRP (Cat. No. A4416; Sigma) diluted 1:1,000 in blocking buffer and developed in hydrogen peroxide/DAB (3.3′-diaminobenzidine) solution.

### Guide RNA Selection and Synthesis

We selected A238L (5EL), a non-essential ASFV gene (Dixon et al., 2004) to assess the possibility of knocking out ASFV gene by CRISPR/Cas9-NHEJ and CRISPR/Cas9-HDR pathways. A guide RNA design tool, CHOPCHOP (Labun et al., 2019), was used to identify and select A238L gene-specific gRNA; AGTAGGCCTGTTTTCAGCCG targeting N-terminus (close to the start codon) of the A238L gene. Precision gRNA synthesis kit (Invitrogen) was used to synthesize the A238L (5EL) gRNA by *in vitro* transcription (IVT) according to the manufacturer’s instruction.

### Plasmid Construction

To delete the A238L (5EL) gene by homology-directed repair (HDR), a plasmid was designed using SnapGene software (GSL Biotech; available at snapgene.com) and ordered from GenScript. The plasmid consists of pUC57-Mini vector background containing the enhanced green fluorescent protein (eGFP) under p72 promoter and 10T thymidylate terminator regulatory elements, flanked by 1,000 bp-long regions homologous to the sequences upstream and downstream of A238L (5EL) gene. The eGFP and the regulatory elements are floxed by LoxP sites for subsequent excision through Cre-Lox recombination. Upon integration into ASFV genome, the complete A238L (5EL) coding region is replaced with the eGFP expression cassette.

### ASFV Infection and Transfection

Cas9-expressing WSL (WSL-Cas9) cells were grown in 24-well plates and maintained in DMEM + 10% FBS + penicillin/streptomycin + glutamine + 1 μg/ml puromycin dihydrochloride at 37°C in a humidified atmosphere and 5% CO_2_.

For A238L(5EL) gene knock out attempt by NHEJ, 24 h later, ASFV-Ke-dsRed virus (Hübner et al., 2018) was added to the wells at multiplicity of infection (MOI) of 0.5 for 4 h at 37°C under 5% CO_2_. After 4 h of virus adsorption, supernatant was removed, and the cells were washed twice with 1 × PBS and replenished with fresh medium. IVT-synthesized A238L gRNA was transfected into the WSL-Cas9 cell line using Lipofectamine MessengerMAX (Invitrogen) as per the manufacturer’s instruction. Forty-eight hours later, the plate containing cells plus medium were frozen at −80°C and the cells were thawed at 37°C.

For A238L gene deletion attempt by HDR, IVT-synthesized A238L gRNA and linear amplicon (eGFP repair template generated from eGFP plasmid using p5/p6 primer pair) were transfected into WSL-Cas9 cell independently infected with wildtype ASFV-Ke and ASFV-Ke-dsRed viruses using Lipofectamine 3000 as per the manufacturer’s manual.

### Genomic Cleavage Detection, Deletion-Screening PCR, and Sanger Sequencing

**FIGURE 1:**
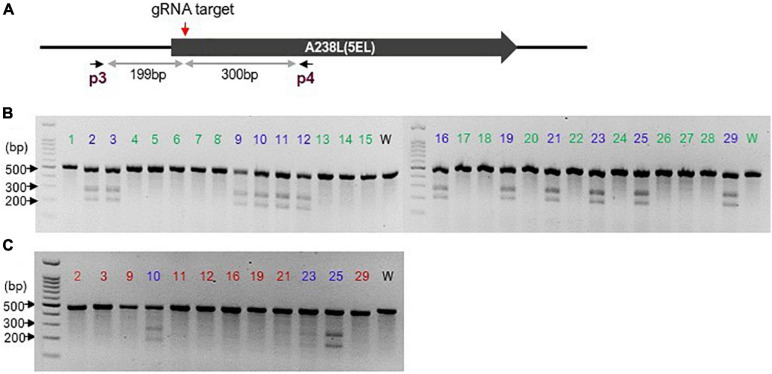
CRISPR/Cas9-mediated genomic cleavage detection assay: **(A)** Schematic showing the gRNA-targeted region (red downward arrow) of the A238L (5EL) gene and the p3p4 primer pairs (flanking the gRNA target site) used to generate 499 bp fragment that would yield two distinct product bands of unequal sizes upon cleavage by the detection enzyme. **(B)** The 499 bp amplicon from analyzed clones (1–29) was spiked with similar amplicon from wildtype (w) ASFV to establish whether two additional distinct bands indicative of genomic cleavage are present. Clones labeled in blue produced mismatched amplicons hence are potentially edited. Clones labeled in green produced uniform amplicons (like wild-type amplicon) hence un-edited. **(C)** clones showing multiple bands in (**B**; labeled in blue) were subjected to further cleavage detection assay to rule out wildtype contamination. Clones (red) showing a single band were deemed to be edited clones. Numbers 1–29 indicates analyzed clones while w is the wildtype control.

ASFV DNA was extracted from the culture using a DNA extraction kit (Qiagen). For the NHEJ approach, the extracted DNA was subjected to the GeneArt^TM^ genomic cleavage detection assay (Invitrogen) to confirm genomic cleavage resulting from Cas9-induced DSB, using a T7 endonuclease I (T7EI) based method. Briefly, a 499-bp of the modified locus was amplified using primers p3 and p4 so that the potential cleavage site was not in the centre of the amplicon hence yielding two distinct product bands upon cleavage by the detection enzyme and resolution on a gel. Upon confirmation of genome modification of the initial transfectants, the virus was subjected to 5 rounds of fluorescence-focused dilution cloning (as described below) to obtain pure modified virus clones. The resulting clones were analyzed for the presence of edits using GeneArt^TM^ genomic cleavage detection kit (Invitrogen) with slight modification, as follows. Individual “clonal” progenies’ DNA was amplified using primers p3 and p4 and the resulting amplicon was spiked with a similar amplicon from wildtype ASFV DNA. This admixture was then subjected to denaturation and re-annealing (at 95°C and gradual reduction in temperature to 25°C, respectively) steps and cleavage with detection enzyme and bands resolved on an agarose gel (Figure 1).

To estimate the on-target genome editing efficiency generated by NHEJ activity, the relative proportion of DNA contained in each band was analyzed using a gel image analysis software, Fiji/ImageJ (Schindelin et al., 2012), and the formulae in the genomic cleavage detection kit (Invitrogen) manual applied.

To determine the cleavage and repair pattern at the targeted locus, amplicons generated with p3/p4 primer pair were purified using High Pure PCR product purification kit (Roche) and shipped to Macrogen Europe B.V. (Amsterdam, Netherlands) for Sanger sequencing with the same primers. The sequences were then analyzed using SnapGene for modification patterns (insertion, deletions, and frame-shift mutation) by comparing to the wildtype sequence.

For the HDR approach, stable integration of the repair template in the targeted genome locus was confirmed by PCR and sequencing. Genomic forward and reverse primers (p9 and p12) were designed using the regions outside the recombination sites. Primers corresponding to the internal sequence of the eGFP cassette were also designed (p10 and p11). Integration of eGFP donor template in ASFV genome was confirmed by amplification from primer pairs p9/p10 (5′ integration) and from p11/p12 (3′ integration). Amplification from p9/p12 was used to discern ΔA238L and wildtype viruses based on size polymorphisms. To confirm the absence of A238L (5EL) in the ΔA238L viruses, primer pairs p7/p8, internal to A238L (5EL), were designed and PCRs were performed on both wildtype and ΔA238L viruses. Additionally, to confirm the stable integration of eGFP in the A238L locus, p9/p12-generated amplicons were subjected to Sanger sequencing reactions using seq1, seq2, seq3, and seq4 primers. The generated nucleotide sequences were aligned against the expected p9/p12-amplicon sequence. All primers used in this study are listed in Supplementary Table 1.

### Isolation of Modified Virus by Fluorescence-Focused Dilution Cloning

To reduce the time to isolate individual ASFV deletion mutants, we combined fluorescence microscopy, cloning by limiting dilution technique, and a Nikon object marker, a technique we refer to as fluorescence-focused dilution cloning. Following successful transfection and confirmation of eGFP integration via PCR and fluorescence microscopy, WSL cells in 24-well plates was infected with serial dilutions of the supernatant of the transfectants and incubated at 37°C and 5% CO_2_. During the incubation, 2% low-melting agarose/DMEM (5% FBS) was melted for 2–3 min in the microwave and the required volume transferred to a 50 ml falcon tube and placed in water bath pre-set at 37°C. After the 2-h incubation, the virus inoculum was removed, and the cells were washed with 1 × PBS. The molten agarose was mixed with equal volume of DMEM/10% FBS to obtain 1% low-melting agarose/DMEM (5% FBS) overlay which was then used to cover the cells. After 3–5 days of incubation, the recombinant viruses’ foci were spotted by the green and/or red fluorescence under an inverted fluorescence microscope (Optika). The fluorescent region of interest was then marked using Nikon object marker low power scanning objective (×10). Briefly, rotating the object marker (fitted into the objective nosepiece of a microscope in place of one of the objectives) into the optical path, the user presses on the knurled ring, and the underside of the plate is left with a 1.8 mm ink circle around the point of interest. The overlayed agar in the marked circle was then gently scooped out and discarded using the opposite end of pipette tips without disturbing the rest of the agar or the confluent cell layer. The cells were then scraped and harvested using 50 μl of fresh DMEM and transferred into sterile 1.5 ml tubes (containing 250 μl of DMEM). The tube was then freeze-thawed at −80°C/37°C and spun down to collect the supernatant. DNA was extracted from 50 μl of the supernatant and subjected to PCR to confirm eGFP integration and purity (absence of the wildtype or parental virus). This cloning and PCR cycle was repeated until the recombinant virus was devoid of wild type virus.

### Next Generation Sequencing of ASFV Genomes and Bioinformatic Analyses

Parental and mutant ASFV genomes were purified and sequenced using the Illumina platform to confirm targeted insertion of eGFP into the desired genome locus, and to screen for possible CRISPR/Cas9-induced off-target genome cleavage and potential genome deletions and re-arrangements, that may have occurred due to passaging the viruses in WSL cells. Briefly, ASFV-Ke-ΔA238L, and ASFV-Ke-dsRed-ΔA238L mutants and their respective parental viruses were independently grown in WSL cells and purified using 36% (w/v) sucrose cushion ultracentrifugation. DNA was then extracted from the enriched virus pellets using Qiagen DNA extraction kit. The extracted DNA was further purified and processed as per Kumar et al. (2020).

Raw FastQ reads obtained were trimmed to remove low-confidence bases using Trimmomatic (release 0.38) (Bolger et al., 2014) with the following parameter settings: LEADING:10; TRAILING:10; SLIDINGWINDOW:4:20; MINLEN:25. Host reads were eliminated by mapping the trimmed reads to the Sus scrofa genome (assembly 11.1) using Bowtie 2 aligner (v2.3.4.1) (Langmead and Salzberg, 2012). *De novo* assembly was generated using Unicycler (v0.4.7) (Wick et al., 2017). To confirm knock-in and knock-out locations, contig sequences were aligned to the genome assembly of ASFV-Ke (Hübner et al., 2018). Polymorphisms between assembled contigs of parent and mutant clones were identified by sequence alignment and confirmed by mapping back the trimmed reads to the contigs. Polymorphisms with read depth of at least 6 and base quality of >20 were accepted as high confidence variants.

### *In vitro* Virus Replication Studies

The *in vitro* replication kinetics of each of the generated deletion mutants and wildtype ASFV were evaluated using quantitative PCR. WSL cells were infected in triplicates with ASFV-Ke, ASFV-Ke-dsRed, ASFV-Ke-ΔA238L, and ASFV-Ke-dsRedΔA238L viruses at an MOI of 0.1. The infected WSL cells were incubated for 4, 24, 48, 72, and 96 h at 37°C under 5% CO_2_, and frozen at each time point at −80°C. Genomic DNA was extracted from thawed lysate using a DNA extraction kit (Qiagen), and qPCR was performed using QuantStudio 5 system (Applied Biosystems, United States). Each reaction was conducted in du-plicates in 20 μl reaction mixture containing 6.46 μl nuclease−free water, 10 μl EXPRESS qPCR Supermix (Invitrogen), 0.6 μl of forward primer (10 μM), 0.6 μl of reverse primer (10 μM), 0.3 μl of TaqMan^®^ probe (10 μM), 0.04 μl of ROX and 2 μl template DNA. Plasmid standard was diluted 10-fold from 10^10^ to 10° copies/μl. Nuclease-free water was used as the negative control. The following qPCR profile was used: 50°C for 2 min, 95°C for 2 min, followed by 45 cycles of 95°C for 15 s and 60°C for 1 min. The primers used for qPCR are listed in Supplementary Table 1.

## Results

### Establishment of Cas9-Expressing WSL Cell Line

To generate a versatile Cas9-expressing cell line which is adaptable to modification of any ASFV gene, we used Lipofectamine 3,000 reagent to transfect WSL cells with the pSpCas9(BB)-2A-Puro (PX459) v2.0 plasmid (Ran et al., 2013). This plasmid bicistronically co-expresses Cas9 and puromycin resistance genes from a single CAG promoter through T2A peptide, and therefore obtaining a puromycin-resistant WSL clone would be an initial proof of expression of the Cas9 nuclease. We chose the WSL cell because it is suitable for productive replication of ASFV (Portugal et al., 2012; Keil et al., 2014; Hübner et al., 2018), and it does not interfere with genome integrity of the investigated ASFV strain (unpublished result), whereas after adaptation of ASFV to Vero cells, large deletions resulting in loss of virulence and protective capabilities were frequently observed (Tabarés et al., 1987; Krug et al., 2015; Rodríguez et al., 2015). Similar deletions and re-arrangements in ASFV genome have also been observed for viruses passaged in PIPEC (Borca et al., 2021) and CV-1 (Mazloum et al., 2019) cells. Prior to transfection, we determined that 1 μg/ml of puromycin dihydrochloride was the suitable concentration for selecting resistant clones using dose-response evaluation. After transfection and drug selection, the resulting puromycin-resistant cells were cloned by limiting dilution and Cas9 expression confirmed in eight of the clones by Western blotting. We re-confirmed Cas9 expression in one of the clones we chose for this study (Supplementary Figure 1). The Cas9-expressing WSL cells exhibited similar growth as the parental line.

### CRISPR/Cas9 Modification of the ASFV Genome via NHEJ Pathway

To demonstrate the utility of CRISPR/Cas9 to edit the ASFV genome, we initially employed the NHEJ pathway to generate targeted knock-out in the ASFV genome. To facilitate the cloning process of NHEJ-modified virus, a fluorescent ASFV expressing dsRed was used (Hübner et al., 2018). The CRISPR/Cas9-mediated DSB genome cleavage is ensued by the activation of cell repair machinery that preferentially inserts or deletes nucleotide(s) at the cleaved genomic location. Unlike HDR, NHEJ is extremely efficient and active throughout the cell-cycle and does not require a homologous repair template. In this study, we used one guide RNA, gRNA (AGTAGGCCTGTTTTCAGCCG) adjacent to the start codon of A238L to maximize the chances of creating a non-functional protein (Figure 1A). To as-certain if a modification has occurred at the specified genome location, we used the Genome Cleavage Detection Kit^TM^ (Invitrogen). This assay involves first amplifying the locus of interest followed by a denaturing and re-annealing step. Re-annealed mismatched DNA heteroduplex strands (from unmodified wildtype and modified DNA strands annealing together) are detected and cleaved by a detection enzyme in the kit, seen as two distinct bands when resolved on a gel, as depicted in Supplementary Figure 2 and Figures 1B,C. Figure 1A shows a schematic of the gRNA-targeted region of the A238L (5EL) gene and the position of primer pairs used to generate a fragment that would yield two distinct product bands of unequal sizes upon cleavage by the detection enzyme.

Following successful CRISPR/Cas9-mediated genome cleavage at the A238L (5EL) locus (Supplementary Figure 2), indicating 14.3% on-target gene editing efficiency as estimated using Fiji/ImageJ (Schindelin et al., 2012), the virus was cloned by five rounds of fluorescence-focused dilution cloning. Twenty-nine clones were individually tested for cleavage and repair using cleavage detection assay and several of these clones have modifications, as shown by the appearance of two bands (Figure 1B) in clonal ASFV DNA spiked with wildtype ASFV DNA due to mismatch between edited and wildtype amplicons. Clones showing a single band after spiking with wildtype ASFV DNA amplicons were interpreted as wildtype ASFV since the amplicons were matching and hence no cleavage by the cleavage detection enzyme. Modified clones showing two bands were analyzed further by another round of cleavage detection assay (without spiking with wildtype DNA amplicon). However, when wildtype virus DNA has not been added (Figure 1C), presence of two bands indicate mismatching by differential editing, or by a mixture of edited and residual wildtype DNA strands, and therefore the presence of two or more virus clones. Nine of the clones, translating to 31% of the analyzed clones, showed a single band (Figure 1C) demonstrating that genomic modification had indeed occurred at the targeted site.

### ASFV Genome NHEJ Repair Has Higher Propensity for In-Frame Deletion

**FIGURE 2:**
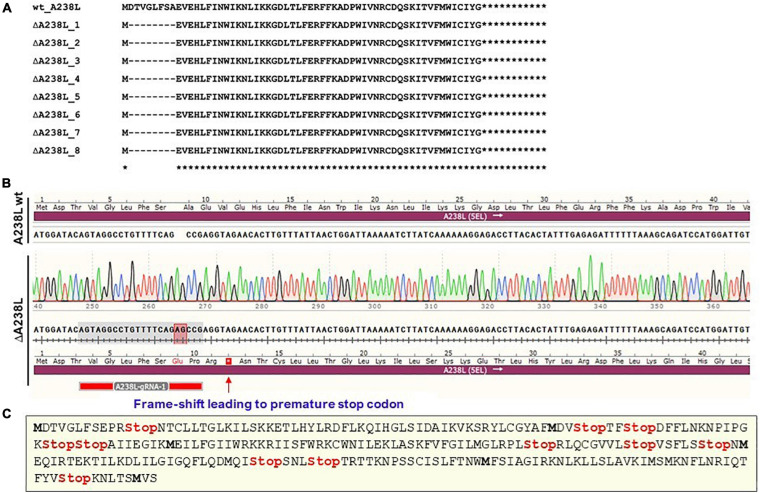
Indels and frameshift mutations: **(A)** Amino acid sequences from edited clones show a consistently uniform 8 amino acid in-frame deletions at the cleavage and repair site in A238L (5EL). **(B)** Two-nucleotide insertion (shadowed red), 3nt upstream of the PAM (NGG) site generating a frame-shift (out-of-frame) mutation hence creating premature stop codons. The target location of the gRNA is shadowed in gray. **(C)** Multiple stop codons (red) and internal methionine codons (bold) generated in the frameshifted A238L (5EL) ORF by the 2nt insertion.

To ascertain the nature of NHEJ-generated indels in the targeted ASFV locus, the nine clones were analyzed by Sanger sequencing. The resulting sequences of the nine clones were processed in SnapGene software, and the nucleotides translated to amino acid sequences using ExPASy Translate tool (Gasteiger et al., 2003). The resulting amino acid sequences were aligned against the 238-amino acid-long wildtype A238L protein using Clustal Omega (EMBL-EBI) (Sievers et al., 2011), which showed a consistently uniform eight amino acid deletion at the cleavage site (Figure 2A) in eight of these clones, representing 88.9% of the edited clones. Interestingly, this deletion was in-frame, generating a potentially semi-functional A238L protein (Figure 2A). Only one clone, constituting 3.4% of the analyzed clones and 11.1% of the modified clones, had an “AG” nucleotides insertion 3nt upstream of the PAM (NGG) site generating the desired frame-shift (out-of-frame) mutation (Figure 2B), hence creating a premature stop codon (and other multiple stop codons) in the A238L ORF effectively yielding a non-functional protein (Figure 2C). This observation illustrates that the preferred out-of-frame deletion occur at much lower frequency as compared to the undesired in-frame deletion and is congruent with previous reports that deletions are much more prevalent than insertions (Bae et al., 2014). Such a tendency of the NHEJ repair pathway to generate in-frame deletions could result in replication-competent escape mutants that are no longer recognized by the specific guide RNAs as observed in Herpesviruses and HIV-1 (van Diemen et al., 2016; Wang G. et al., 2016; Wang Z. et al., 2016), making this approach unsuitable for generating deletion mutants. Nevertheless, this undesirable in-frame deletion can be circumvented by using combinatorial approach of multiple gRNAs against a specific ASFV gene to enhance complete knock-out.

### CRISPR/Cas9 Modification of the ASFV Genome via HDR Pathway

The HDR pathway employs a homologous donor sequence to accurately insert exogenous pieces of DNA into a genome following DNA DSB by CRISPR/Cas9. This approach has pioneered a range of applications for inserting entire genes (e.g., fluorescent proteins), generating or correcting SNPs, creating precise indels, and inserting epitope tags for studying gene functions as well as creating genetically modified organisms.

**FIGURE 3:**
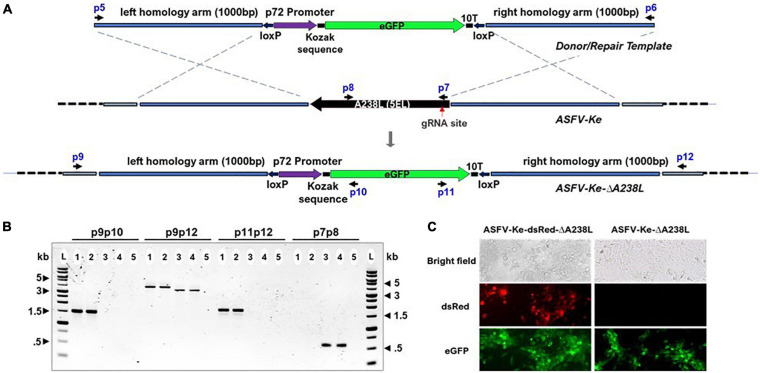
Schematic and confirmation of the A238L (5EL) knockout strategy in ASFV-Ke and ASFV-Ke-dsRed. **(A)** Linear PCR amplicon encoding enhanced green fluorescence protein (eGFP), under the control of p72 promoter and 10T thymidylate terminator, flanked by 1,000 bp-long regions homologous to the sequences upstream and downstream of A238L (5EL). Upon integration, the complete A238L (5EL) coding region is replaced with the eGFP cassette. Black arrows represent primers: p5 and p6 used to linearize the eGFP plasmid; p9, p10, p11, and p12 to confirm successful knockout of A238L; and p7 and p8 to confirm the absence of the A238L locus in ΔA238L viruses. p9 and p12 are external forward and reverse primers outside the recombination sites while p10 and p11, reverse and forward primers, respectively, are in-ternal to eGFP gene and are used in combination with external primers to confirm A238L (5EL) deletion. Amplification from primer pair p9/p10 confirms 5′ integration and amplification from primer pair p11/p12 confirms 3′ integration of eGFP cassette in the A238L (5EL) locus. The p9/p12 amplification discriminates ΔA238L (3.7 kb) from wildtype (3.3 kb) based on band sizes. **(B)** Agarose gel showing PCR screening using different primer combinations on ASFV-Ke-ΔA238L (1), ASFV-Ke-dsRedΔA238L (2), ASFV-Ke wt (3), ASFV-Ke-dsRed (4), and no-template control (5) confirming the successful integration of the eGFP cassette replacing the A238L (5EL) gene and showing that ΔA238L viruses are devoid of the wildtype A238L(5EL) gene. **(C)** Microscopic fluorescence images showing eGFP-expressing and dsRed-expressing ASFV-infected WSL cells.

To modify the ASFV genome by HDR, we generated a plasmid harboring a repair template containing the enhanced green fluorescent protein (eGFP) surrounded by 1,000 bp-long regions homologous to the sequences upstream and downstream of A238L (5EL) gene (Figure 3A) to facilitate efficient homologous recombination effectively deleting A238L (5EL). To facilitate subsequent removal of exogenous DNA through Cre-Lox re-combination, eGFP and the regulatory elements are flanked by LoxP sites. This approach is geared toward generating a relatively “cleaner” gene edit devoid of exogenous DNA, e.g., fluorescent markers or for sequential deletion of multiple genes (Abrams and Dixon, 2012).

Stable integration of the repair template in the targeted genome locus was confirmed by PCR amplification (Figure 3B) and Sanger sequencing (Supplementary Figure 3). The amplification from primer pairs p9 and p10 generated a 1,488-bp product confirming the 5′ integration, and from p11 and p12 with a 1,681-bp product confirming the 3′ integration of eGFP donor amplicon in the ΔA238L viruses (Figure 3B). The amplification from p9 and p12 generates products of 3,740 and 3,337 bp in ΔA238L and wildtype viruses, respectively (Figure 3B). The amplification from primer pairs p7 and p8, internal to A238L (5EL) (Figure 3A), confirms the absence of A238L (5EL) in the ΔA238L viruses while a single 572-bp band was detected in the parental ASFV-Ke wt and ASFV-Ke-dsRed ASFVs (Figure 3B). Sanger sequencing of ΔA238L p9/p12-generated amplicons with seq1, seq2, seq3, and seq4 primers confirmed targeted insertion of eGFP in the A238L locus (Supplementary Figure 3). Figure 3C shows microscopic fluorescence images confirming the expression of eGFP in ASFV-Ke-ΔA238L and ASFV-Ke-dsRed-ΔA238L infected cells.

### Isolation of Recombinant Virus by Fluorescence-Focused Dilution Cloning

To isolate individual eGFP-expressing ASFV ΔA238L deletion mutants, a combination of fluorescence microscopy, limiting dilutions, and a Nikon object marker was used. Marking the fluorescent region of interest using the object marker increases the yield and isolation of the fluorescent recombinant. The attainment of a pure recombinant clone was verified through PCR after every cloning cycle. With this simple approach, five rounds of modified cloning by limiting dilution were sufficient for obtaining fluorescent mutants devoid of wildtype ASFV contaminations, thereby significantly reducing the time to obtain recombinant viruses to <2 months.

### Off-Target Cleavage Analysis and Confirmation of Targeted eGFP Integration

Illumina MiSeq^TM^ complete genome sequencing was employed to determine that only the desired modifications were introduced into the genome of the recombinant ASFV-Ke-ΔA238L and ASFV-Ke-dsRed-ΔA238L viruses. On average 6.8 million reads were obtained per sample. After filtering out low quality reads on average 2.2 million read pairs per sample mapped to ASFV genome (see Supplementary Table 2 for exact numbers). An average read depth of 1,436 × was obtained per sample (Supplementary Table 2 and Supplementary Figure 4). Assembled contigs of mutant clones were compared against their parental clones to screen for any off-target effects of CRISPR/Cas9 on the mutant genome, targeted integration of eGFP, and genome deletions and re-arrangements that may have arisen as a result of passaging the viruses in WSL cells. No off-target mutations or deletions were observed in ASFV-Ke-ΔA238L mutants compared to their parent. Only one SNP (TΔC) was present in a non-coding region (position 37623) in ASFV-Ke-dsRed-ΔA238L. The read depth and alternate allele frequency at this locus were 130 and 100%, respectively, indicating the SNP was called with high confidence. Additionally, the absence of deletions, rearrangements, and significant SNPs in these sequenced genomes is an indication that WSL does not interfere with genome integrity of the investigated ASFV.

Through *de novo* genome assemblies generated from the Illumina reads we confirmed the targeted deletion of A238L and insertion of eGFP in its place for both ASFV-Ke-ΔA238L and ASFV-Ke-dsRed-ΔA238L mutants (Supplementary Figures 5A,B).

### *In vitro* Replication Kinetics of Wildtype and Mutant ASFVs

**FIGURE 4:**
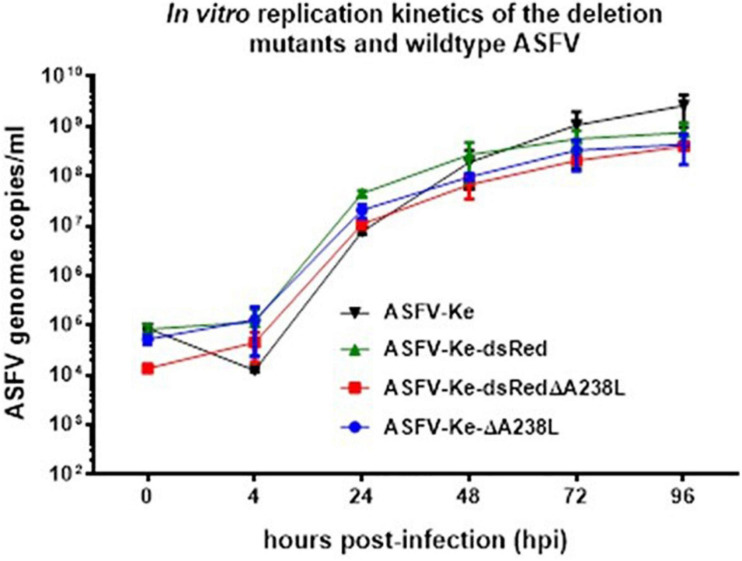
*In vitro* replication kinetics of parental and mutant ASFVs: WSL cells were infected with the indicated viruses at an MOI of 0.1 and viral genome copies measured at 0, 4, 24, 48, 72, and 96 hpi. Data represent average of three independent infections and error bars represent the standard deviations.

To compare the *in vitro* replication kinetics of the wildtype and mutant ASFVs, individual viruses were infected at MOI of 0.1 in WSL cells and viral genome copies measured at different timepoints throughout the infection using qPCR. Comparisons of the resulting ASFV genome copies show that the deleted A238L gene did not slow down the growth of the mutant viruses (Figure 4), as has been reported to be non-essential for the *in vitro* replication of Pol18/28298/Out111 genotype II (Woźniakowski et al., 2020) and Malawi Lil-20/1 genotype XIII (Neilan et al., 1997b) isolates. This consensus observation is in line with the high conservation of A238L (5EL) among different ASFV isolates (Neilan et al., 1997b).

## Discussion

In this study, we have provided a detailed description of the HDR approach for editing the ASFV genome. We also explored the practicality of generating ASFV deletion mutants through the NHEJ approach, thus shedding light on the nature and frequency of in-frame deletion mutations observed in the latter. Previous use of CRISPR/Cas9/NHEJ approach in modification of the A238L gene has not been successful since no genome modification was evident after sequencing (Woźniakowski et al., 2020). Our work makes a significant improvement since, by targeting the same gene as Woźniakowski et al. (2020), we have shown successful modification through both NHEJ and HDR, isolated the mutants and characterized the indel patterns. However, in the NHEJ approach, we observed that the preferred out-of-frame indel occurred infrequently as opposed to in-frame deletion since only 11.1% of the modified clones had nucleotides insertion whereas 88.9% of the edited clones had consistent eight amino acid deletion. This observation is consistent with a previous report that deletions are much more prevalent than insertions (Bae et al., 2014). Therefore, while the NHEJ may be attractive since there is no need for donor DNA template and recombination events, it turns out to be unsuitable due to frequent in-frame deletions. Additionally, since there is usually no marker gene in this approach, all the modified clones need to be sequenced to identify the preferred out-of-frame indel. In the wider perspective, an ASF vaccine based on an indel-based gene knockout may also be a riskier strategy than deleting the whole gene by recombination or a dual guide-RNA strategy, as it may more easily revert to virulence without the need of recombination. However, had the NHEJ pathway produced plenty of out-of-frame indels, it could have been a faster way to generate ASFV mutants for functional screening, which could then be followed by production of the full gene deletion mutants through the HDR pathway. In conclusion, the HDR pathway is the method of choice for generating modified ASF viruses, with the option of deleting the fluorochrome(s) in the final viruses of choice to avoid the presence of foreign DNA in the vaccine viruses.

Using NGS, we have confirmed the targeted insertion of eGFP in the genome (consequently the deletion of A238L gene) and the purity (absence of wildtype contamination) in the mutant ASFV compared to the parental viruses. Additionally, NGS data on the parental and mutant ASFVs replicating in WSL and WSL-Cas9 cells did not show significant modifications resulting from either Cas9 off-target cleavage or passaging the virus. No off-target mutations or deletions were observed in ASFV-Ke-ΔA238L mutants compared to their parent. Only one single nucleotide polymorphism (SNP) present in a non-coding region was observed in ASFV-Ke-dsRed-ΔA238L and was absent in the parent clone. Additionally, the absence of SNPs, deletions, and re-arrangements in these sequenced genomes is an indication that WSL may not interfere with genome integrity of the investigated ASFV. This is in contrast to previous reports where progressive adaptation of ASFV to Vero, PIPEC and CV-1 cell lines resulted in large deletions and re-arrangements in ASFV genome (Tabarés et al., 1987; Krug et al., 2015; Rodríguez et al., 2015; Mazloum et al., 2019; Borca et al., 2021).

We also describe an efficient protocol to use for fast cloning of modified ASF viruses. In our protocol, agarose was used for overlaying the cells before picking of the fluorescent foci. Several protocols use a viscous methylcellulose medium (Hübner et al., 2019) for their cloning steps, which is easier to handle, but inhibits spread of released virus a bit less efficiently. Apart from the combined limiting dilution, fluorescence microscopy and object marking, the agarose may therefore contribute to less contamination with wildtype virus during the cloning steps. The timeframe for obtaining a deletion mutant in this study was < 2 months. In contrast, up to 3–4 months have been reported for single gene deletions (Rathakrishnan et al., 2020) and ten or more cloning rounds have been reported using standard homologous recombination with conventional plaque purification techniques (Borca et al., 2020). Our timeframe is consistent with the approach employed by Rathakrishnan et al. (2020), which combines the conventional homologous recombination with fluorescent-activated cells sorting (FACS) to isolate and purify viruses expressing fluorescent reporter genes. However, our approach circumvents the need for the expensive cell sorters making it suitable for use in resource-limited settings.

Taken together, our detailed description of the HDR approach for editing the ASFV genome combined with an efficient and fast protocol for cloning the modified viruses should aid in facilitating expeditious generation of ASFV vaccine candidates by the scientific community.

## Data Availability Statement

The data has been deposited to NCBI, Bioproject PRJNA747972.

## Author Contributions

HA, NS, NA-G, WF, SV, and LS: conceptualization. HA, NS, BO, EA, SH, SO, SM, and LS: methodology. HA and SH: formal analysis and data curation. HA, LS, SV, NS, SH, SO, NA-G, WF, BO, EA, and SM: investigation. LS and SV: resources and funding acquisition. HA: writing—original draft preparation. HA, NS, WF, LS, SH, and SV: writing—review and editing. HA, BO, EA, and SM: visualization. LS: supervision and project administration. All authors have read and agreed to the published version of the manuscript.

## Conflict of Interest

The authors declare that the research was conducted in the absence of any commercial or financial relationships that could be construed as a potential conflict of interest.

## Publisher’s Note

All claims expressed in this article are solely those of the authors and do not necessarily represent those of their affiliated organizations, or those of the publisher, the editors and the reviewers. Any product that may be evaluated in this article, or claim that may be made by its manufacturer, is not guaranteed or endorsed by the publisher.
