# Supplementary material for: Rapid CRISPR/Cas9 Editing of Genotype IX African Swine Fever Virus Circulating in Eastern and Central Africa
Source: Front Genet. 2021 Aug 30;12:733674. doi: 10.3389/fgene.2021.733674 (PMC8435729; doi:10.3389/fgene.2021.733674)
Supplement: Supplementary file 1 [file Data_Sheet_1.docx]

Supplementary Material

## Supplementary Figure 1.


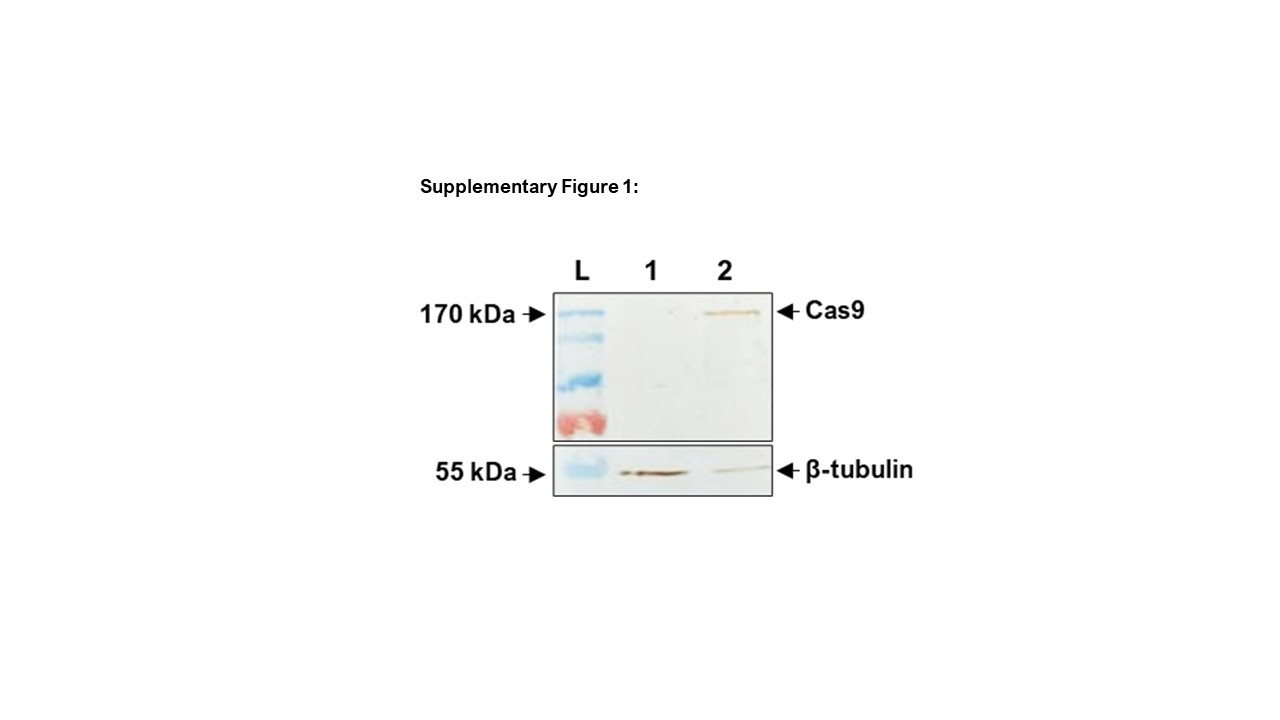


**Western blot analysis of Cas9 expression:** Expression of Cas9 was detected by Western blot in WSL-Cas9 cells (WSL cells transfected with pSpCas9(BB)-2A-Puro (PX459) plasmid) [lane 2], but not in the wildtype WSL [lane 1]. A parallel blot incubated with a β-tubulin specific monoclonal antibody was used as loading control. Equal amount (10µg) of proteins were loaded on the SDS-PAGE gel well. The expected 161 kDa Cas9 protein and 55 KDa β-tubulin is indicated by an arrow. Molecular masses of marker proteins (L) are indicated.

## Supplementary Figure 2.


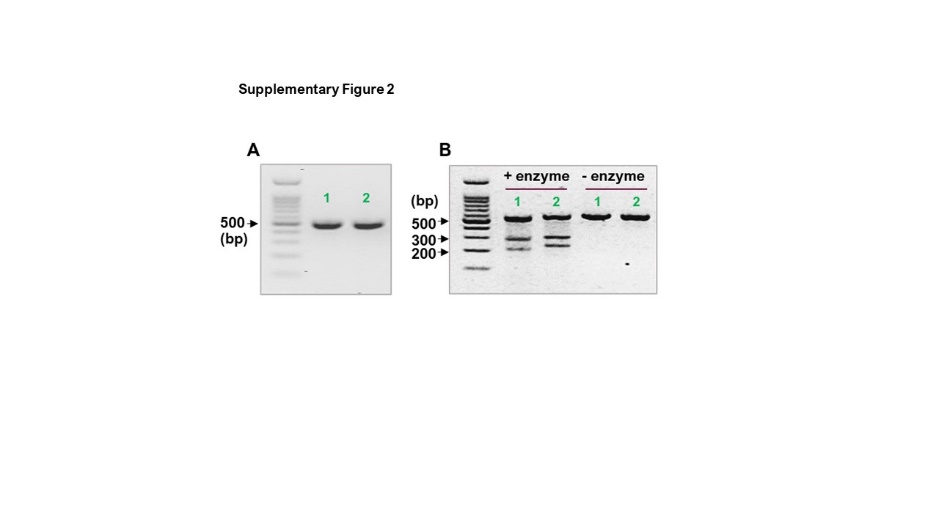


**CRISPR/Cas9-mediated genomic cleavage detection assay**: a) 499bp fragment generated by primer pairs p3/p4 flanking the A238L (5EL) gRNA target site. b) Re-annealed mismatched DNA heteroduplex strands (from unmodified wildtype and modified DNA strands) are detected and cleaved by a detection enzyme showing successful genome cleavage. 1 and 2 represent the A238L amplicon and control template, respectively, with or without detection enzyme.

## Supplementary Figure 3.


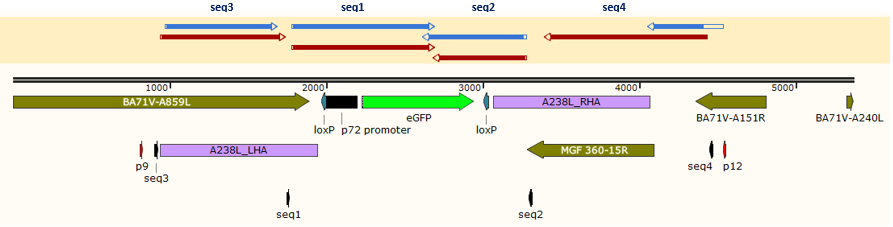


**Confirmation of eGFP integration in A238L locus in ASFV-Ke-∆A238L and ASFV-Ke-dsRed∆A238L mutants by Sanger sequencing:** Alignment of sequences of ∆A238L p9/p12 amplicons (Sanger-sequenced with seq1, seq2, seq3, and seq4 primers) against the expected sequence confirmed targeted insertion of eGFP in the A238L locus. The maroon and blue top arrows indicate aligned sequences generated for ASFV-Ke-∆A238L and ASFV-Ke-dsRed∆A238L mutants, respectively. The alignments were generated using SnapGene software.

## Supplementary Figure 4.


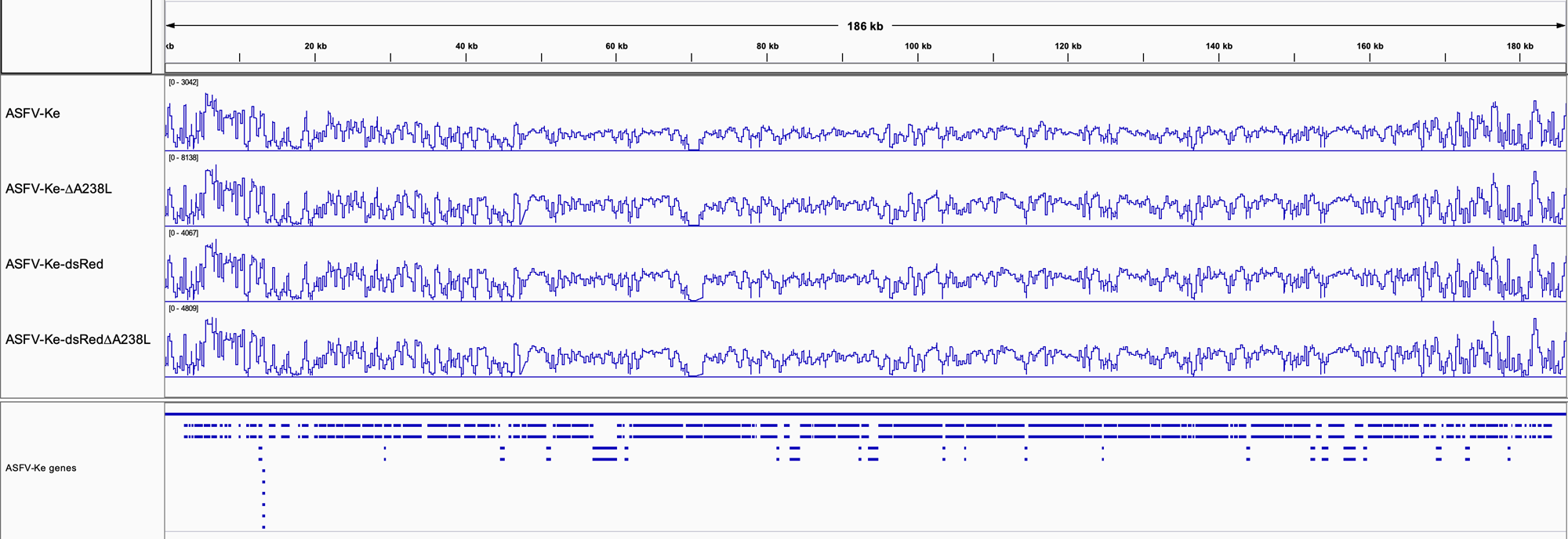


**Genome depth:** Depth across the genome is shown in blue lines for the four samples sequenced. Mean depth for ASFV-Ke = 811**×**, ASFV-Ke-∆A238L = 1351**×**, ASFV-Ke-dsRed = 2157**×**, and ASFV-Ke-dsRed∆A238L = 1426**×**. Read depth boundaries for each sample are shown in square brackets above each sample track. Gene distribution in the genome is shown as blue rectangles in the bottom panel.

## Supplementary Figure 5A.


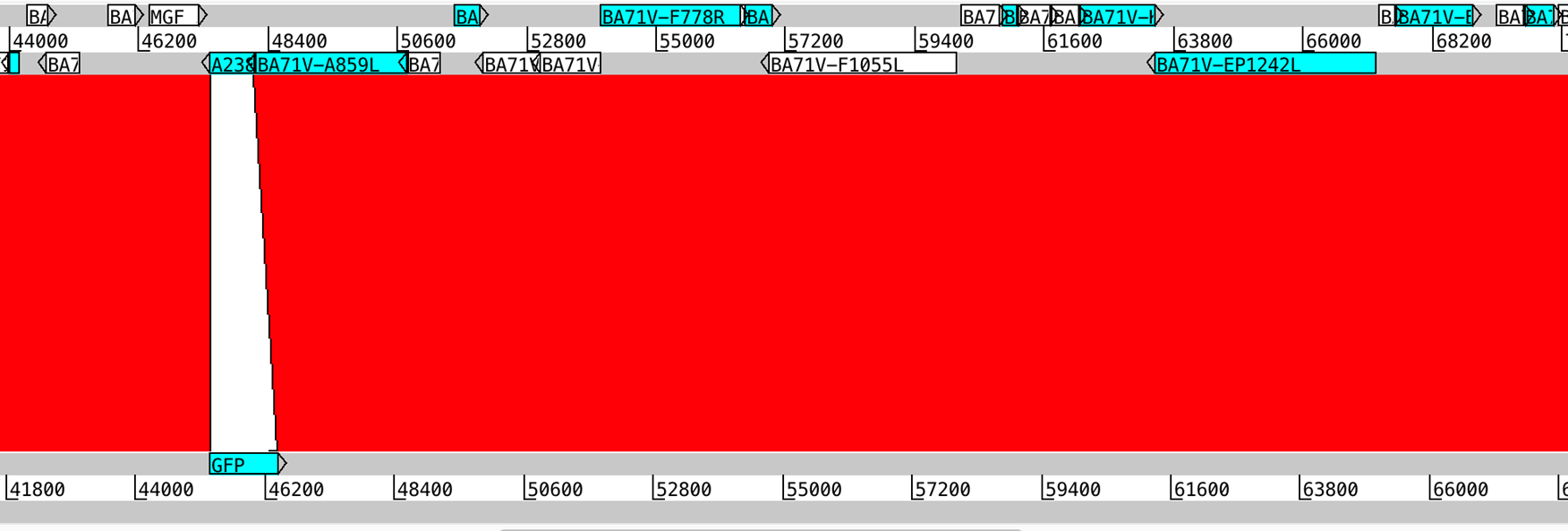


## Confirmation of A238L replacement with eGFP in ASFV-Ke-∆A238L mutant: ASFV-Ke genome (top panel) is compared with the *de novo* assembly of ASFV-Ke-∆A238L (bottom panel). The middle red panel marks regions with sequence similarity between the two genomes, which was 99% in the segments flanking the modified loci. The white space indicates there is no sequence similarity in ASFV-Ke-∆A238L at gene A238L. Furthermore, the A238L locus in ASFV-Ke-∆A238L is replaced by eGFP, as highlighted by the annotation in turquoise rectangles. Figure generated using Artemis Comparison Tool.

## Supplementary Figure 5B.


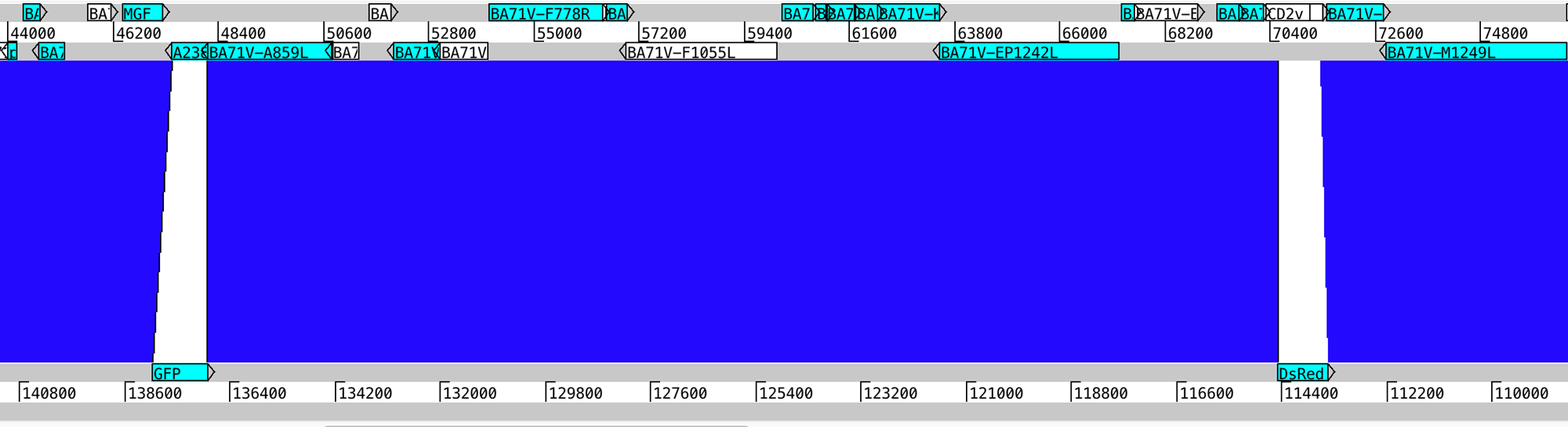


**Confirmation of A238L replacement with eGFP in ASFV-Ke-dsRed∆A238L mutant:** ASFV-Ke (top panel) compared with the *de novo* assembly of ASFV-Ke-dsRed∆A238L (bottom panel). The white space in the middle indicates the absence of A238L and CD2v genes and insertion of GFP and dsRed (turquoise box). Blue lines in the middle show regions of nucleotide sequence similarity between the two genomes, which was 99% in the segments flanking the modified loci. Figure generated using Artemis Comparison Tool.

**Supplementary Table 1: Primers used in this study.**

| **Name** | **Sequence** | **Description** |
| --- | --- | --- |
| p1 | TAATACGACTCACTATAGAGTAGGCCTGTTTTCAGCCG | Forward primer for generating A238L gRNA by in vitro transcription |
| p2 | TTCTAGCTCTAAAACCGGCTGAAAACAGGCCTACT | Reverse primer for generating A238L gRNA by in vitro transcription |
| p3 | TCCCCGGGATAGGATTCTTGT | Forward primer for detecting genomic cleavage of A238L (5EL) gene |
| p4 | GCCCACCTGTATTCAAGAAGC | Reverse primer for detecting genomic cleavage of A238L (5EL) gene |
| p5 | ATGATGGTACCCGGGGATCC | Forward primer for generating linear donor/repair eGFP template |
| p6 | GCTCACATGTTCTTTCCGATGG | Reverse primer for generating linear donor/repair eGFP template |
| p7 | ATGGATACAGTAGGCCTGTTTTCAG | Forward primer for confirming deletion of A238L in ∆A238L ASFV genome |
| p8 | GGGAAATGCATGAAGAGGGGT | Reverse primer for confirming deletion of A238L in ∆A238L ASFV genome |
| p9 | AGGCCGCTGAACAAGCAG | Forward primer for screening 5′ integration of the donor template in ASFV genome |
| p10 | CGTTTACGTCGCCGTCCAG | Reverse primer for screening 5′ integration of the donor template in ASFV genome |
| p11 | GCGCGATCACATGGTCCT | Forward primer for screening 3′ integration of the donor template in ASFV genome |
| p12 | GGTGAACCGCGAATTCGC | Reverse primer for screening 3′ integration of the donor template in ASFV genome |
| seq1 | ACTGGCCTGGTTATGCCAAAG | Sequencing primer for confirming eGFP integration in A238L locus |
| seq2 | GCTCGAGATTGCAATCTTACGC | Sequencing primer for confirming eGFP integration in A238L locus |
| seq3 | AGTATTATCCCCTTTGCAGACGC | Sequencing primer for confirming eGFP integration in A238L locus |
| seq4 | AGGTTTCAACCTCAGTTGGTCCT | Sequencing primer for confirming eGFP integration in A238L locus |

**Supplementary Table 2: NGS metrics summary.**

| **Sample** | **Raw reads in pairs** | **Total reads passed quality filter** | **Reads mapped to ASFV-Ke** | **Average depth** |
| --- | --- | --- | --- | --- |
| ASFV-Ke | 6,557,220 | 5,355,318 | 1,231,224 | 811 |
| ASFV-Ke-dsRed | 6,985,630 | 4,600,625 | 2,027,307 | 1,351 |
| ASFV-Ke-∆A238L | 7,836,404 | 7,053,788 | 3,408,568 | 2,157 |
| ASFV-Ke-dsRed-∆A238L | 6,155,438 | 5,258,661 | 2,113,184 | 1,426 |
| *Average* | *6,883,673* | *5,567,098* | *2,195,071* | *1,436* |
